# $W^+_{} W^-_{} H$ production at lepton colliders: a new hope for heavy neutral leptons

**DOI:** 10.1140/epjc/s10052-018-6279-x

**Published:** 2018-09-29

**Authors:** J. Baglio, S. Pascoli, C. Weiland

**Affiliations:** 1 Institut für Theoretische Physik, Eberhard Karls Universität Tübingen, Auf der Morgenstelle 14, 72076 Tübingen, Germany; 2 Institute for Advanced Study, Durham University, Cosin’s Hall, Palace Green, Durham, DH1 3RL UK; 3 Department of Physics, Institute for Particle Physics Phenomenology, Durham University, South Road, Durham, DH1 3LE UK

## Abstract

We present the first study of the production of a Standard Model Higgs boson at a lepton collider in association with a pair of *W* bosons, $e^+_{} e^-_{} \rightarrow W^+_{} W^-_{} H$, in the inverse seesaw model. Taking into account all relevant experimental and theoretical constraints, we find sizable effects due to the additional heavy neutrinos up to $-38\%$ on the total cross-section at a center-of-mass energy of 3 TeV, and even up to $-66\%$ with suitable cuts. This motivates a detailed sensitivity analysis of the process $e^+_{} e^-_{} \rightarrow W^+_{} W^-_{} H$ as it could provide a new, very competitive experimental probe of low-scale neutrino mass models.

## Introduction

Neutrino oscillations, as discovered by the Super-Kamiokande experiment in 1998 [1] and subsequently confirmed by a plethora of results [2], imply that at least two neutrinos have a non-zero mass. This cannot be explained in the Standard Model (SM) and calls for an extension of this framework. One of the simplest possibilities is the addition of new fermionic gauge-singlet states that play the role of right-handed neutrinos, leading to the type-I seesaw mechanism and its variants [3–18]. Amongst the various seesaw realizations, one of particular interest is the inverse seesaw model (ISS) [10–12]. It was proved in [19] that, in any model that only adds fermionic gauge singlets to the SM field content with no cancellation between the contributions to the light neutrinos masses from different orders of the seesaw expansion or different radiative orders, requiring the light neutrinos to be massless is equivalent to requiring lepton number to be conserved. The inverse seesaw verifies all these conditions and we indeed observe that in the lepton-number-conserving limit of this model, light neutrinos are massless, independently from the seesaw scale or the size of the neutrino Yukawa coupling. In this renormalizable, testable, low-scale seesaw model, light neutrino masses are suppressed not by a small-active sterile mixing as in the high-scale type I seesaw. Instead, this model relies on an approximately conserved lepton number in agreement with the theorem [19], thus allowing to generate the light neutrino masses while having large neutrino Yukawa couplings and heavy sterile neutrinos at the TeV scale, opening the exciting possibility of detecting the latter in current or future planned high-energy colliders, see for example Refs. [20–22] for reviews. It is particularly worth noting that this model provides a prototype of fermionic low-scale seesaw, making our results applicable to a wide range of models.

As the neutrino Yukawa couplings in the ISS can be large, the properties of the Higgs boson, the remnant of the electroweak symmetry-breaking mechanism [23–26] generating the masses of the other fundamental particles in the SM and that was discovered at the Large Hadron Collider (LHC) in 2012 [27, 28], can be sizeably affected. This opens new search strategies which rely on the Higgs boson, for instance Higgs decays [29–32], searches in Higgs production at lepton colliders [33, 34], or lepton flavour violating Higgs decays [35, 36]. We also investigated recently the heavy neutrino impact on the triple Higgs coupling [37, 38].

Based on the idea that *t*-channel fermions coupled to a Higgs boson can give sizeable contributions to a cross-section, see for example the case of $b\bar{b}\rightarrow W^+_{} W^-_{} H$ at the LHC [39], we investigate in this paper, for the first time, the impact of heavy neutrinos on the production of a Higgs boson in association with a pair of *W* bosons at a lepton collider, $e^+_{} e^-_{} \rightarrow W^+_{} W^-_{} H$. This process has been studied in the SM and has been found to have good detection prospects [40]. We describe the ISS model and discuss the relevant theoretical and experimental constraints. We present our calculational setup before a numerical analysis of our results is carried out. Performing a scan over the relevant parameters of the model, we find deviations up to $-38\%$ on the total cross-section at 3 TeV, that can be enhanced to $-66\%$ after applying a reasonable set of cuts that leaves an ISS cross-section of 0.14 fb. We also provide a simplified formula which reproduces our results within one percent.

## The model and its constraints

The ISS model [10–12] is an appealing low-scale seesaw model that extends the SM with fermionic gauge singlets. We consider here a realisation where each generation is supplemented with a pair of these right-handed gauge singlets, $\nu _R^{}$ and *X*, which have opposite lepton number. This provides a realistic realisation of seesaw models close to the electroweak scale that can reproduce low-energy neutrino masses and mixing while being in agreement with all experimental bounds. The additional mass terms to the SM Lagrangian are

1 $$\begin{aligned} \mathscr {L}_\mathrm {ISS} = - Y^{ij}_\nu \overline{L_{i}} \widetilde{\varPhi } \nu _{Rj} - M_R^{ij} \overline{\nu _{Ri}^C} X_{Rj} - \frac{1}{2} \mu _{X}^{ij} \overline{X_{i}^C} X_{j} {+} \mathrm { h.c.},\nonumber \\ \end{aligned}$$

where $\varPhi$ is the SM Higgs field and ${\widetilde{\varPhi }}=\imath \sigma _2 \varPhi ^*$, $i,j=1\dots 3$, $Y_\nu$ and $M_R$ are complex matrices and $\mu _{X}$ is a complex symmetric matrix. A major characteristic of the ISS is the presence of a naturally small lepton-number-breaking parameter $\mu _{X}$ to which the light neutrino masses are proportional. Indeed after block-diagonalising the full neutrino mass matrix, the $3\times 3$ light neutrino mass matrix is given by [41]

2 $$\begin{aligned} M_{\mathrm {light}} \simeq m_D M_R^{T-1} \mu _X M_R^{-1} m_D^T, \end{aligned}$$

at leading order in the seesaw expansion parameter $m_D M_R^{-1}$, where $m_D=Y_\nu \langle \varPhi \rangle$. This decouples the light neutrino mass generation from the mixing between active and sterile neutrinos (that is proportional to $m_D M_R^{-1}$) and allows for large Yukawa couplings even when the seesaw scale is close to the electroweak scale. It is worth noting that in this model, the heavy neutrinos form pseudo-Dirac pairs where the splitting is controlled by $\mu _X$ as can be seen from diagonalizing the 1-generation neutrino mass matrix, which gives

3 $$\begin{aligned} m_{N_1,N_2}=\pm \sqrt{M_R^2+m_D^2} + \frac{M_R^2 \mu _X}{2(M_R^2+m_D^2)}, \end{aligned}$$

in the seesaw limit $\mu _X \ll m_D, M_R$ [36].

Since one of the main motivations of our model is to explain neutrino oscillations, we reproduce low-energy data from the global fit NuFIT 3.0 [42] by using the $\mu _X$-parameterisation adapted to include next-order terms in the seesaw expansion that are relevant for large active-sterile mixing [38]

$$\begin{aligned} \begin{aligned} \mu _X \simeq&\left( \mathbf {1}-\frac{1}{2} M_R^{*-1} m_D^\dagger m_D M_R^{T-1} \right) ^{-1}\, \\&\times M_R^T m_D^{-1} U_{\mathrm{PMNS}}^* m_\nu U_\mathrm{PMNS}^\dagger m_D^{T-1} M_R\, \\&\times \left( \mathbf {1}-\frac{1}{2} M_R^{-1} m_D^T m_D^* M_R^{\dagger -1}\right) ^{-1}\,. \end{aligned} \end{aligned}$$

$m_\nu$ is the diagonal light neutrino mass matrix and $U_{\mathrm{PMNS}}$ is the unitary Pontecorvo-Maki-Nakagawa-Sakata (PMNS) [43, 44] that diagonalises $M_{\mathrm {light}}$. We have chosen $\delta =0$ for the Dirac CP phase in $U_{\mathrm{PMNS}}$ for simplicity. We fix the lightest neutrino mass to 0.01 eV, in agreement with the Planck results [45]. The strongest experimental constraints for this study come from a global fit [46] to electroweak precision observables (EWPO), tests of CKM unitarity and tests of lepton universality. Since we choose all mass matrices and couplings in the neutrino sector to be real and consider diagonal Yukawa couplings $Y_\nu$ in our study, we do not expect electric dipole moment measurements and lepton-flavour-violating processes to provide relevant constraints in this scenario. Finally, we require that the Yukawa couplings $Y_\nu$ remain perturbative, namely

4 $$\begin{aligned} \frac{|Y_{ij}|^2}{4 \pi } < 1.5\,. \end{aligned}$$

## Calculational details

The cross-section is calculated at leading order (LO), both in the SM and in the ISS. Next-to-leading order electroweak corrections have been calculated in the SM [47] and are found to be negligible for center-of-mass (c.m.) energies above 600 GeV and of the order of $-2\%$ at $\sqrt{s}=500$ GeV, that would correspond to the lowest International Linear Collider c.m. energy that would be relevant for our process [48]. Given the size of the ISS deviation we obtain (of the order of $-5\%$ at $\sqrt{s}=500$ GeV and down to $-38\%$ at higher c.m. energies, see later), we will not take these electroweak corrections into account in our analysis.

**Fig. 1:**
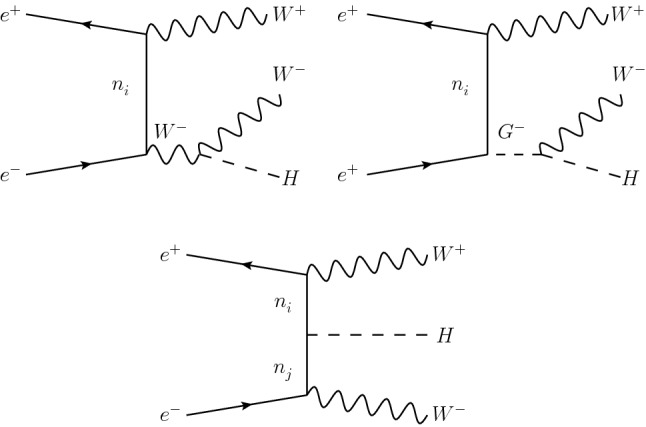
Generic Feynman diagrams representing the ISS neutrino contributions to $e^+_{}e^-_{}\rightarrow W^+_{} W^-_{} H$ in the Feynman-’t Hooft gauge. The indices *i*, *j* run from 1 to 9. Mirror diagrams are obtained by flipping all the electric charges

The charged leptons are taken massless and their coupling to the Higgs boson is neglected. The calculation is done in the Feynman-’t Hooft gauge. The Feynman diagrams at LO include *s*–channel exchanges of a *Z* boson or a photon, as well as *t*–channel diagrams involving the neutrinos for which a generic selection is displayed in Fig. 1. The remaining *t*–channel diagrams are obtained with a flipping of the *W* and charged Goldstone boson contributions from the $W^-_{}$ line to the $W^+_{}$ line. We have used our own ISS model file developed for the packages FeynArts 3.7 and FormCalc 7.5 [49, 50] to generate a Fortran code, and the numerical integration has been performed with BASES 5.1 [51] in order to obtain a selection of kinematic distributions.

Similar to the SM calculation, the interference terms are significant and destructive. The dominant contribution to the ISS amplitude comes from the first two diagrams in Fig. 1 with heavy neutrinos and which go as $|Y_\nu |^2 v^2/M_R^2 (a+b v^2/M_R^2)$, and from the third diagrams with one heavy neutrino and one light neutrino in the *t*–channel which goes as $|Y_\nu |^2 v^2/M_R^2$, in terms of the seesaw parameters. In order to enhance the cross-section we have also performed a calculation with polarised beams. More specifically, we have chosen, based on the Compact Linear Collider (CLIC) baseline [52], an unpolarised positron beam, $P_{e^+_{}}^{} = 0$, and a polarised electron beam with $P_{e^-_{}}^{}=-80\%$. If we define $\sigma _{LR(RL)}^{}$ as the cross-section for a completely polarised left-handed (right-handed) positron with $P_{e^+_{}}^{} = -100\% (+100\%)$ and a completely polarised right-handed (left-handed) electron with $P_{e^+_{}}^{}=+100\%(-100\%)$, the polarised cross-section for arbitrary polarisation fractions $P_{e^+_{}/e^-_{}}^{}$ can be written as [53]

5 $$\begin{aligned} \sigma _{\mathrm{pol}}^{} = \frac{1}{4}\Big [&(1-P_{e^+_{}}^{})(1+P_{e^-_{}}^{}) \sigma _{LR}^{} + (1+P_{e^+_{}}^{})\nonumber \\ {}&\left. (1-P_{e^-_{}}^{})\sigma _{RL}^{}\right] , \end{aligned}$$

since the LL and RR cross-sections are identically zero in our process.

## Numerical results

The calculation is done in the $G_\mu ^{}$ scheme (see e.g. Ref. [54], and Ref. [55] in the context of neutrino mass models) and the input parameters are the *Z* mass $M_Z^{}$, the *W* boson mass $M_W^{}$ and the Fermi constant $G_F^{}$. Including the Higgs mass $M_H^{}$, the parameter values are chosen as

6 $$\begin{aligned} M_W^{}&= 80.385~\text {GeV}\,,\,\, M_Z^{} = 91.1876~\text {GeV}\,,\nonumber \\ M_H^{}&= 125~\text {GeV}\,,\,\, G_F^{} = 1.16637\times 10^{-5}_{}~\text {GeV}^{-2}_{}\,. \end{aligned}$$

Based on our previous analysis on the triple Higgs coupling [38], we use the $\mu _X^{}$-parameterisation with a degenerate Yukawa texture, $Y_\nu ^{} = |Y_\nu ^{}| I_3^{}$, with hierarchical heavy neutrinos, $M_R^{} = \text {diag}(M_{R_1^{}}^{}, M_{R_2^{}}^{},M_{R_3^{}}^{})$. To illustrate our results we select the same hierarchy as in [38],

7 $$\begin{aligned} M_{R_1^{}}^{} = 1.51 M_R^{},\,\, M_{R_2^{}}^{} = 3.59 M_R^{},\,\, M_{R_3^{}}^{} = M_R^{}. \end{aligned}$$

From now on, $M_R^{}$ is to be understood as a number as well in a slight abuse of notation. These specific heavy neutrino mass ratios are related to our choice of $Y_\nu =|Y_\nu ^{}| I_3^{}$ since they make the constraints of the global fit [46] impact every generation similarly.

**Fig. 2:**
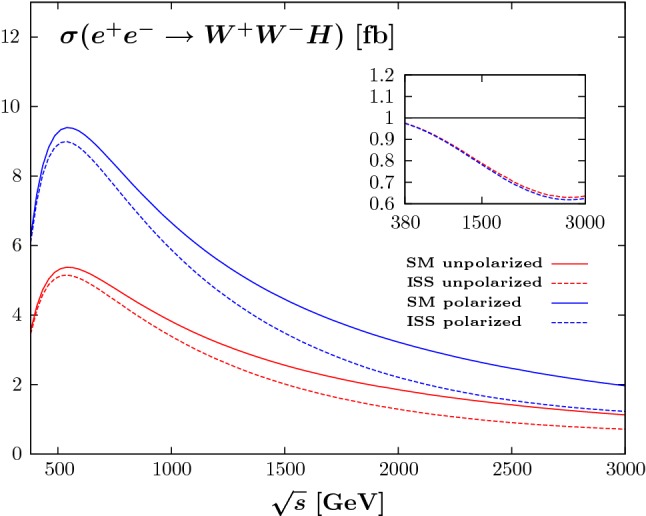
LO total $W^+_{}W^-_{} H$ production cross-section at an electron-positron collider (in fb) as a function of the c.m. energy $\sqrt{s}$ (in GeV). The solid curves stand for the SM predictions, the dashed curves stand for the ISS predictions using the benchmark scenario described in the text. The red (blue) curves are for an unpolarised ($-80\%$ polarised electron beam) cross-section. The ratio of the ISS cross-section with respect to the SM prediction is shown in the insert

We present in Fig. 2 the variation of the total production cross-section $\sigma (e^+_{}e^-_{}\rightarrow W^+_{}W^-_{}H)$ as a function of the c.m. energy $\sqrt{s}$, using a benchmark scenario with $|Y_\nu ^{}| = 1$ and $M_R^{} = 2.4$ TeV, resulting in a heavy neutrino spectrum with three pairs of pseudo-Dirac neutrinos of mass 2.4, 3.6, and 8.6 TeV. We stress that this scenario, with reasonable $\mathscr {O}(1)$ Yukawa couplings, is allowed by current experimental and theoretical constraints.

The gain by going from an unpolarised cross-section to the polarised electron beam is illustrated by the factor-of-two difference between the red curves (unpolarised) and the blue curves (polarised). The behaviour of the ISS contribution in the polarized cross-section is the same as that of the unpolarized one, meaning that the use of a polarized beam will lead to more events thus increasing the sensitivity to the large deviations coming from the ISS. The maximum of the cross-section is obtained at c.m. energies around 500 GeV, for which the ratio of the ISS cross-section with respect to the SM cross-section, shown in the insert, is around 0.95. The negative contribution from the ISS correction increases with higher c.m. energies, reaching already $20\%$ for $\sqrt{s}\sim 1.4$ TeV and a maximal deviation of $-38\%$ at a c.m. energy close to 3 TeV, from which the ISS correction starts to decrease for increased c.m. energies.

**Fig. 3:**
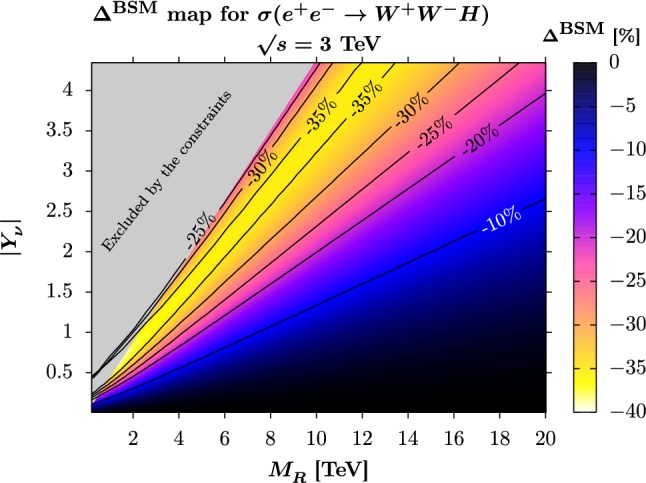
Contour map of the neutrino corrections $\varDelta _{}^\mathrm{BSM}$ (in percent) to the $W^+_{}W^-_{} H$ production cross-section at a 3 TeV electron-positron collider, using a $-80\%$ polarised electron beam, as a function of the seesaw scale $M_R^{}$ (in GeV) and $|Y_\nu ^{}|$ in the $\mu _X^{}$-parameterisation, using a diagonal Yukawa texture and a hierarchical heavy neutrino mass matrix with the parameters defined in Eq. (7). The grey area is excluded by the constraints

In order to get insights into the dependence of the ISS correction on the parameters of the ISS, we have performed in Fig. 3 a scan of the ISS deviation with respect to the SM production cross-section, $\varDelta ^{\mathrm{BSM}}_{} = (\sigma ^{\mathrm{ISS}}_{}-\sigma ^\mathrm{SM}_{})/\sigma ^{\mathrm{SM}}_{}$, as a function of the seesaw scale $M_R^{}$ and of the parameter $|Y_\nu ^{}|$ for the diagonal Yukawa texture we have chosen and still using heavy hierarchical neutrinos with the parameters of Eq. (7). The c.m. energy is fixed to $\sqrt{s} = 3$ TeV which is the last stage of the CLIC baseline, with a $-80\%$ polarised electron beam. The grey area is excluded by the constraints applied to the ISS, the global fit to EWPO and low-energy data [46] being the dominant constraint.

The ISS contribution vanishes, as expected, for a large seesaw scale $M_R^{}$ and for vanishing Yukawa couplings. For a large fraction of the parameter space, deviations of at least $-20\%$ are allowed, and they reach a peak of $-38\%$, interestingly for Yukawa couplings $|Y_\nu ^{}|\sim 1$ and a seesaw scale of a few TeV. The ISS deviation is then decreasing when approaching the region of excluded points, reaching $\varDelta ^{\mathrm{BSM}}_{} = -25\%$. Using our previous analysis of the dependence on the seesaw parameters, we have devised the following approximate formulae to reproduce $\varDelta ^{\mathrm{BSM}}$ in the region allowed by the experimental constraints and for $M_R>3$ TeV,

8 $$\begin{aligned} \mathscr {A}^{\mathrm{ISS}}_{\mathrm{approx}} =\,&\frac{(1~\text {TeV})_{}^2}{M_R^2} \mathrm{Tr} (Y_\nu ^{} Y_\nu ^\dagger )\, \left( 17.07 - \frac{19.79~\text {TeV}_{}^2}{M_R^2}\right) ,\nonumber \\ \varDelta ^{\mathrm{BSM}}_{\mathrm{approx}} =\,&(\mathscr {A}^{\mathrm{ISS}}_{\mathrm{approx}})_{}^2 -11.94\, \mathscr {A}^\mathrm{ISS}_{\mathrm{approx}}. \end{aligned}$$

The coefficients (calculated here for $\sqrt{s} = 3$ TeV) depend on the kinematics of the process and in particular on the c.m. energy. We have checked that, for $M_R^{} > 3$ TeV, our fit reproduces the full result within $1\%$ in the region where the numerical error of our calculation is negligible. For $M_R^{} < 3$ TeV, higher-order terms in $1/M_R^{}$ that we have not included for simplicity and clarity give sub-leading corrections that degrade the agreement between our fit and the full calculation. For example, we find for our benchmark scenario with $|Y_\nu ^{}| = 1$ and $M_R^{} = 2.4$ TeV, the fit deviates from the full result by $6\%$ only. Beside, below $M_R^{}<1.8$ TeV in the allowed region, the fit is off the full results by around $\pm 10\%$ or more and we advise not to use it: We get for example for $|Y_\nu ^{}| = 0.7$ and $M_R^{}=1.8$ TeV the exact result $\varDelta ^\mathrm{BSM}_{} = -38.4\%$ to be compared to the result of our fit $\varDelta ^{\mathrm{BSM}}_{\mathrm{approx}} = -34.8\%$, that is a $9\%$ difference. Compared to a similar map we derived in [38] using the triple Higgs coupling, the coverage with significant deviations is much larger.

**Fig. 4:**
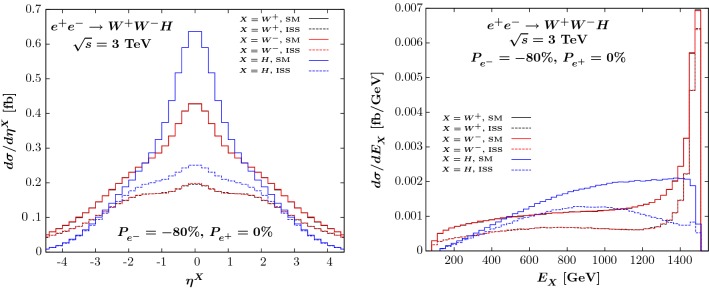
Pseudo-rapidity (left) and energy (right) distributions of the $W^+_{}$ (black), $W^-_{}$ (red) and Higgs (blue) bosons in the process $e^+_{}e^-_{}\rightarrow W^+_{}W^-_{}H$ at a c.m. energy of 3 TeV, using a $-80\%$ polarised electron beam. The solid curves stand for the SM predictions, the dashed curves stand for the ISS predictions using the benchmark scenario described in the text

We have also considered kinematic distributions, in particular the energy and pseudo-rapidity distributions of the final-state particles. They are presented in Fig. 4 in the benchmark scenario we have already chosen for Fig. 2 using Eq. (7). The solid curves represent the SM predictions while the dashed curves stand for the ISS distributions. The $W^+_{}$ (in black) and $W^-_{}$ (in red) distributions are identical for both the pseudo-rapidity (left) and the energy (right) observables, while the Higgs distributions are displayed in blue.

For both $W^\pm _{}$ and Higgs boson, the pseudo-rapidities in the central region have a different behaviour in the SM and in the ISS. More specifically, the ISS corrections are substantial in the region $|\eta |<1$. In the case of the energy spectrum, depicted on the right-hand side of Fig. 4, the ISS correction is distributed over the whole range for the $W^\pm _{}$ bosons, while it starts to be more significant above 1 TeV for the Higgs boson. We have then considered the following two cuts on the cross-section, in order to enhance $\varDelta ^\mathrm{BSM}_{}$: $|\eta _{H/W^\pm _{}}^{}| < 1$ and $E_H^{} > 1$ TeV. Starting from polarised cross-sections $\sigma _{\mathrm{pol}}^\mathrm{SM} = 1.96$ fb and $\sigma _{\mathrm{pol}}^{\mathrm{ISS}} = 1.23$ fb, giving $\varDelta ^{\mathrm{BSM}}_{} = -38\%$, we obtain the polarised cross-sections $\sigma _{\mathrm{pol,~cuts}}^{\mathrm{SM}} = 0.42$ fb and $\sigma _\mathrm{pol,~cuts}^{\mathrm{ISS}} = 0.14$ fb, resulting in $\varDelta ^{\mathrm{BSM}}_{} = -66\%$. This could potentially enlarge the region of interest for the parameter space. We have also checked that the same type of enhancement holds for another choice of the parameter point in the region where $|Y_\nu ^{}| \sim 4$ and $M_R^{} = 8.3$ TeV. Using the same set of cuts we get a deviation of $-34\%$ instead of $-26\%$ for the cross-section without cuts. The level of enhancement is reduced compared to the benchmark scenario with $|Y_\nu ^{}|=1$ because of the shape of the ISS $\eta$ distributions which is closer to that of the SM prediction.

## Summary and outlook

In this article we have investigated the effects of heavy neutrinos on the production of a pair of *W* bosons in association with a Higgs boson at a lepton collider, $e^+_{}e^-_{}\rightarrow W^+_{}W^-_{} H$. After taking into account the constraints on the model we have found sizeable deviations that are maximal at a c.m. energy of 3 TeV corresponding to the last stage of the CLIC baseline, reaching a 38% decrease of the cross-section with respect to the SM prediction, in regions of the parameter space with Yukawa couplings $|Y_\nu ^{}|\sim 1$ and a seesaw scale of a few TeV. Analysing the kinematic distributions, we have found that the negative deviations can be enhanced when using suitably chosen cuts on the cross-section and reach $-66\%$. This is the first time the effects of an extended neutrino sector on the production cross-section of a pair of *W* bosons in association with a Higgs boson at a lepton collider have been investigated and our results highlight the potential of this observable to beat future LHC measurements which lose sensitivity in the high mass regime [22]. They also demonstrate the ability of this process to probe the coupling to the Higgs boson which is common to all see-saw type I and III and their extensions, and motivate a detailed sensitivity analysis of $e^+_{}e^-{}\rightarrow W^+_{}W^-_{} H$ [56] as this could provide a new, very competitive, and complementary observable to probe neutrino mass models, especially in $\mathscr {O}(10)$ TeV mass regimes with diagonal and real $Y_\nu ^{}$ that are difficult to probe otherwise.
